# Concordant GRADE-3 Truncal Ataxia and Ocular Laterodeviation in Acute Medullary Stroke

**DOI:** 10.3390/audiolres13050068

**Published:** 2023-10-18

**Authors:** Jorge C. Kattah

**Affiliations:** College of Medicine, Neurology University of Illinois, Peoria, IL 61637, USA; kattahj@uic.edu

**Keywords:** acute vestibular syndrome (AVS), severe truncal ataxia (STA), HINTS, ocular laterodeviation (OLD), lateral medullary stroke (LMS), Wallenberg’s syndrome, Vertigo

## Abstract

**Background:** Severe truncal ataxia associated with an inability to sit up without assistance (STA grade 3) is frequent in patients with central acute vestibular syndrome (AVS) involving the brainstem or cerebellum. When these patients have nystagmus, central HINTS excludes peripheral lesions; however, additional localization and lateralization signs are helpful, not only to resolve the peripheral versus central vestibular lesion dilemma, but to zero in on a precise lesion localization/lateralization to the lateral medulla, the most common ischemic lesion localization associated with an initially false-negative stroke MRI. **Methods:** This is a study of AVS patients with additional inclusion criteria: grades 2 or 3 ataxia with an eventual diagnosis of medullary stroke (MS), either involving the lateral medulla (LMS) or the medial medulla (MMS), and horizontal (h) gaze paralysis was the main exclusion criteria. All patients sat on the side of the bed or stretcher, with assistance if needed. A general neurologic examination followed in the sitting position, the testing protocol included the head impulse, spontaneous nystagmus, and skew deviation (HINTS) tests, followed by observation of the effect of brief 3–5 sec eyelid closure on ocular position, and saccade and pursuit eye movement tests. If they could sit, the protocol included the ability to stand with a wide base, then a narrow base, the Romberg test, and tandem gait. Radiographic lesion localization and horizontal gaze deviation concluded the protocol. **Results:** A total of 34 patients met the entry criteria, 34 MS (13 in the lateral medulla, 12 previously described, and 1 new patient), and 1 new MMS. Among them, *n* = 10/12 had grade 3 ataxia, and 3 (1 new patient) had grade 2 ataxia. In addition, overt ocular laterodeviation (OLD) was present in thirteen of them (35.3%). All OLD patients had gaze deviation and ipsilateral saccade and truncal lateropulsion, 1 medial medulla stroke patient had grade 3 truncal contrapulsion and contralateral hemiparesis without OLD, n = 20/21 patients with LMS without OLD had grade 3 truncal ataxia, and 1 had grade 2 truncal ataxia. **Discussion:** AVS patients with severe truncal ataxia (inability to sit without assistance) potentially have brainstem, cerebellum, or subcortical lesions. All patients had central HINTS; however, simultaneous direction-concordant STA 3 and OLD provided greater lateral medulla localization specificity, affecting the ipsilateral medulla. Future work to explore a practical posterior circulation stroke scale that includes HINTS, STA, and OLD will potentially select cases for thrombolysis even in the event of initially false-negative imaging.

## 1. Introduction

Acute ischemic lesions of the lateral medulla cause Wallenberg’s syndrome. Typically, they present with heterogeneous clinical findings; however, a substantial number of patients display characteristic localizing findings [1]. Among them, severe truncal ataxia (STA) associated with an inability to sit without assistance is frequent [2,3]. When nystagmus is present, these patients often have central HINTS; therefore, the question of central versus peripheral localization is settled; however, this is the most frequent stroke associated with an initially false-negative MRI [4,5]. Here, additional signs such as maximal ocular laterodeviation (OLD) following brief 3–5 s eyelid closure [6] provide further impetus to obtain repeat imaging [7]. While similar degrees of truncal ataxia occur with other lesions involving the brainstem [8,9] or cerebellum, the combination of direction-concordant OLD and substantial truncal ataxia (grades 2–3) is specific for lateral medulla localization; its sensitivity, however, is low. Interestingly, the pathophysiology of STA and OLD involves a different mechanism. The nuclei responsible for STA include the lateralvestibulospinal tract (LVST), the dorsal spinocerebellar tract (DSCT), or both [10]. These nuclei are adjacent to the origin of the inferior cerebellar peduncle (ICP), and OLD results from the interruption of fibers originating in the inferior olive and traveling through the contralateral ICP on their way to the ocular motor vermis (OMV) [11,12]. As a result, one frequently finds both signs in LMS patients. In addition to the clinical signs outlined above, early radiographic findings precede, in some cases, the appearance of positive diffusion-weighted (DWI) MRI [13]. The management of the central AVS requires precise lesion localization, as the work-up, management, and monitoring of the brainstem, cerebellar, or combined brainstem/cerebellar stroke varies significantly with lesion localization in the posterior fossa. While the focus of this research was on correct diagnosis, the next immediate research goal is to recognize patients who require neurovascular intervention. Here, the NIH stroke scale (NIHSS), by design appropriate for anterior circulation stroke, is not adequate when applied to patients with LMS; thus, future development and implementation of a brainstem ischemic index is necessary. The general prognosis of LMS for independent ambulation is favorable; however, when associated with simultaneous cerebellar or additional brainstem strokes, there is risk for significant posture and gait impairment. In addition, thrombolysis in posterior fossa stroke outside of basilar artery occlusion [14] is limited, and the development of posterior fossa perfusion studies to select cases in need of thrombolysis is in its infancy [15]. 

## 2. Methods

This is a prospective study of high-grade truncal ataxia (grade 2–3) and OLD tested among 161 consecutive AVS patients; this study concerns only those patients with eventual imaging evidence of ischemic stroke in the medulla defined as positive DWI signal involving the medulla. All patients were evaluated at the Illinois Neurologic Institute, Saint Francis Medical Center in Peoria, Illinois between 2004 and 2022. The study was approved by the IRB and followed the principles of the Helsinki Convention. The data are from an ongoing study of stroke in AVS patients. The prospective AVS cohort with high-grade ataxia (2–3) and an eventual diagnosis of medullary stroke were the main inclusion criteria; previous reports outlined the clinical details from 32 patients [6]. All patients sat on the side of the bed or stretcher, with assistance if needed, and a general neurologic examination followed in the sitting position. The protocol first included analysis of the head impulse, spontaneous nystagmus, and skew deviation (HINTS) tests, followed by tests of saccade and pursuit eye movements, and observation of the effect of brief 3–5 s eyelid closure on ocular position. If they could sit, the following incremental posture sequence was observed, depending on ability: standing with a wide base, then a narrow base, the Romberg test, and tandem gait. An inability to sit without assistance (Supplementary Materials Video S1), scored grade 3 [3] OLD, is a complete ocular lateral deviation noted immediately upon the opening of the eyes (i.e., the far lateral aspect of the corneal limbus is “buried” without sclera visible between the limbus and lateral canthus). Finally, the review of the MRI included lesion localization of the predicted acute ischemic lesion. If the first MRI was negative, compelling central vestibular findings prompted a repeat MRI in an average of 48 h. The patients enrolled in this study were examined several times during the first 24 h to identify stroke evolution changes in ocular motor, vestibular, and neurological findings. To measure the degree of radiographic gaze deviation, the method previously described in all our AVS patients [6] in T2 axial MRI scans was utilized. Several clinical examples of OLD in this cohort are available at https://collections.lib.utah.edu/details?id=1407493 (accessed on 2 January 2019). A total of 33 patients in this series underwent follow-up physical examination 1 month after the stroke, 2 patients died at the hospital within 2 weeks after the stroke from unrelated medical complications.

## 3. Results

**Figure 1:**
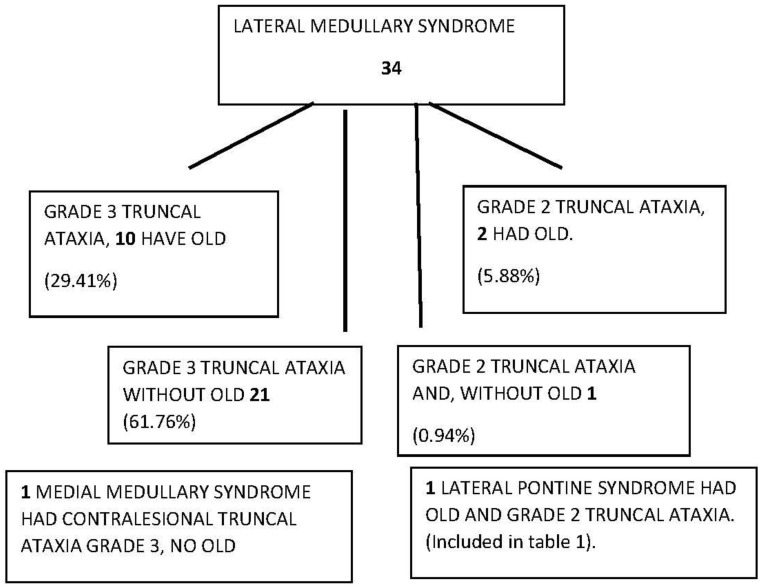
Diagram of truncal ataxia type 3 and OLD laterodeviation in 161 ~24–72 h post-AVS patients.

**Figure 2:**
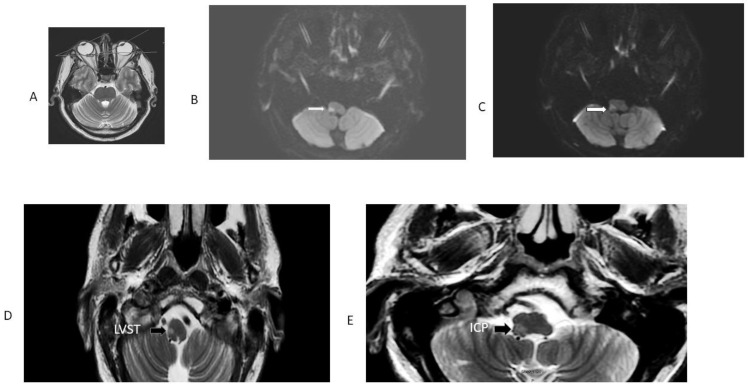
MRI patient 9, Table 1. This patient had an initially false-negative MRI, the Figure shows the repeat MRI 48 h after initiation of symptoms. Note radiographic conjugate horizontal deviation of the eyes toward the right, in the same direction of OLD in a T2 axial MRI (panel (**A**)). Panel (**B**) is an axial DWI; it shows restricted diffusion involving the right LVST and the CSCT, responsible for severe right-side truncal and limb ataxia. The adjacent panel (**C**) is a 3 mm rostral axial DWI section at the level of the right inferior cerebellar peduncle, which explains OLD. The ADC map confirmed diffusion restriction. Panels (**D**) and (**E**) are T2 FLAIR images showing the same structures with greater anatomic resolution.

**Figure 3:**
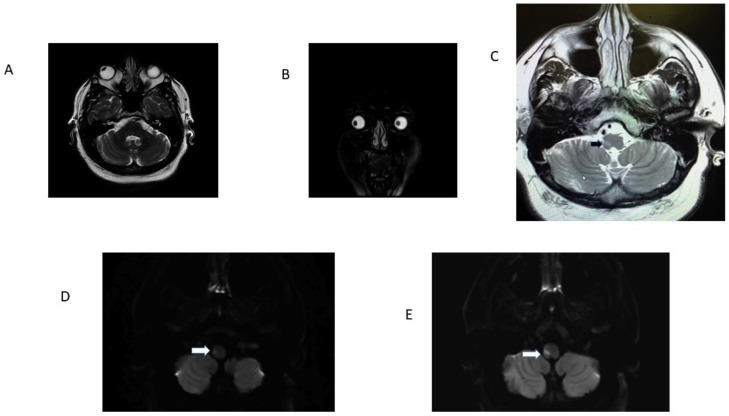
Patient 13 Table 1. Panel (**A**): note radiographic conjugate right ocular deviation, it correlated with clinical right OLD; panel (**B**) is a coronal T2-weighed MRI. Observe radiographic rightward deviation of the eyes in the coronal plane. Panel (**C**) is a T2 FLAIR image that identifies the precise location of the ischemic stroke (black arrow). Panels (**D**) and (**E**) are adjacent axial DWI scans. Note infarcts involving the right LVST and ICP responsible for OLD (white arrows). The ADC map confirmed diffusion restriction.

To begin with, all AVS patients with an eventual medullary stroke had central HINTS. In addition, Table 1 and Figure 1 summarize the findings in the 13 OLD patients (12 lateral medulla and 1 lateral pons). OLD correlated with ipsilesional radiographic gaze deviation and concordant STA; only two OLD patients had grade 2-truncal ataxia, and one of them had a lesion in the pons, included only to illustrate how STA occurs with lesions other than the lateral medulla. Follow-up at 3 months showed resolution of STA in 11 patients; the lateral pontine lesion patient died. One larger lateral medulla stroke (patient 9, Video S1) affected the LVST, DSCT, and ICP (Table 1, Figure 2). His bedside neurological examination performed one week after admission shows STA and a near fall from his wheelchair. In addition, there was right upper extremity ataxia, due to combined LVST and DSCT lesions. Supplementary Materials Video S2 shows his long-term follow-up examination seven years later; he has only mild improvement, currently with severe grade 2 ataxia; he stands and walks with a very wide base and requires a cane. In this series, an initial false-negative MRI of the medulla occurred in four patients (patients 5, 6, 7, 9, Table 1). A repeat MRI obtained 48 h later, utilizing the same MRI stroke protocol and scanner, confirmed the localization/lateralization predicted by the clinical findings. All the patients with OLD had radiographic evidence of horizontal conjugate deviation in the first MRI [16] (Figure 3). None of the patients in this study underwent thrombolysis, and none developed basilar artery thromboses. Vascular imaging in most instances was limited to MRA using different protocols over the length of the study. Patient 1 (Table 1) had an arterial dissection and developed a subsequent pseudoaneurysm that required catheter angiography and surgical repair. The six-month prognosis in this cohort was good for independent ambulation, and thirty-three LMS patients, regardless of OLD/STA or their initial presentation in general, were able to walk without an assistance device.

## 4. Discussion

The systematic organized approach to the correct diagnosis of an AVS has been the subject of focused attention and identifies useful bedside central localizing signs [7]. Lesions in the lateral medulla often cause AVS, theoretically because vestibular nuclei neurons are more susceptible to ischemia than other medullary nuclei and tracts [17]. The vestibular findings appear early and precede other typical localizing findings. It is common to find non-vestibular signs in the first hours after the onset of ischemia/stroke. Examination of the HINTS plus protocol [18] with central characteristics, along with combined OLD and STA (also known as grade 3 truncal ataxia or incubitus ataxia) [2], are additional early signs that localize to the lateral medulla. In a recent single report featuring an AVS patient, OLD and STA occurred in association with an ipsilateral stroke in the lateral medulla (Spielberg, et al.) [19], highlighting the utility of this combination. In this cohort, 35.39% of patients had grade 2–3 STA and OLD. We did not find this combination in peripheral and other locations of central AVS patients. There is a variable combination of the dangerous “D’s”: diplopia, dysphagia, dysarthria, dysmetria, Horner’s syndrome, and pain and temperature sensory loss occurring in the first hours after stroke onset, which are more readily identified as the intensity of the central vestibular findings improve [20,21]. OLD, since the first case series description in 1974, is a highly localizing lateral medulla sign [22,23,24,25,26,27].

To highlight the most important points, I will approach the discussion on selective pathophysiological characteristics, mechanisms, and potential management of ischemic strokes in the medulla.

### 4.1. Presumed Pathogenesis of OLD and STA

The presumed mechanism of OLD involves disruption of the normal straight-ahead horizontal eye position, which requires symmetric activity between brainstem pre-oculomotor circuits including the vestibular and gaze-holding networks, the ocular motor vermis (OMV), and the fastigial ocular motor nucleus (FOR) of the cerebellum [12]. The function of neurons in the FOR is affected indirectly by brainstem lesions, for example, the inferior olive (IO) in LMS; thus, interruption of input to the OMV affects the function of the FOR. Moreover, experimental lesions affecting one side of the OMV more than the other produce a contralateral position bias. Finally, experimental, transient unilateral inactivation of the FOR causes an ipsilateral position bias, like what we found in the OLD patients (Video S1: https://collections.lib.utah.edu/details?id=1407493 accessed on 2 January 2019).

Thomke F. et al. identified severe truncal ataxia with lesions involving the LVST, which was the common structure affected; involvement of the DSCT may also cause truncal ataxia, and compromise of both structures may cause severe truncal ataxia [10]. Damage to the DSCT was clinically detected by ipsilateral limb ataxia, which is not present with LVST lesions. In LMS, isolated severe truncal ataxia may occur [10]. Examples of concurrent, isolated truncal ataxia and OLD without nystagmus were not present in this cohort, and, to my knowledge, there are no reports of isolated severe truncal ataxia and OLD. In this series, three patients with isolated truncal ataxia did not have OLD (not listed in Table 1). Patient 9 is an example of concurrent STA, OLD, and central HINTS. He eventually had a lateral medulla infarct affecting the LVST, DSCT, and ICP; these additional findings, from the start, show upbeat nystagmus with fixation and skew deviation on presentation (Figure 2). He had right facial hypoesthesia and contralateral left limb hypoesthesia. Of note, his first MRI resulted in a false-negative, and showed a lateral medulla stroke forty-eight hours later. Now, seven years later, his gait ataxia is severe, and he can only stand and walk with a wide base (Video S2). Of note, in an autopsy study of one patient with LMS and STA without OLD, who died from an unrelated cause, the patient initially underwent a peripheral head impulse test, but there was no histologic evidence of ischemia in the medial vestibular nucleus (MVN) or perihypoglossal nucleus (PHN), either by observing ischemic changes in neurons or glia. Moreover, the perineural nets in the MVN and PHN were normal. This suggests that there was transient ischemia of the dorsal medial vestibular nucleus [28]. One important fact to keep in mind is the potential involvement of the dorsal medulla in association with both LMS and medial medullary strokes as well.

### 4.2. Temporal Profile of the Evolving Ischemic Stroke Involving the Medulla

A recent publication from the Vascular Committee of the Bárány Society proposed a classification of spontaneous, continuous AVS, based on its temporal characteristics. Assessment of vestibular and neurological signs and symptoms at the 24-hour mark provides key diagnostic information [29]. Within this period, this cohort’s nystagmus intensity frequently lessened substantially, the STA remained unchanged, but OLD became quite distinct. Of note, all medullary lesion patients with OLD had hypometric corrective saccades; in contrast, the patient with a pontine lesion and OLD had one single corrective saccade (https://collections.lib.utah.edu/details?id=1407493 accessed on 2 January 2019). OLD eventually resolves in n = 12/13; one resolved within six months, and one patient (patient 12, Table 1) has had persistent OLD for the last two years.

### 4.3. Neuroimaging of Medullary Strokes 

One pertinent point to emphasize in our LMS series is the urgency to diagnose a stroke in the lateral medulla, as some patients have associated cerebellar strokes [1]. Wallenberg described occlusion of the posterior inferior cerebellar artery (PICA) as the potential cause; however, Miller Fisher later identified vertebral artery (VA) occlusion as the most common cause of LMS, an observation confirmed later in a large LMS series [30,31]. Wallenberg’s syndrome is an example of a lacunar-sized stroke (2–15 mm in size) caused frequently by a large artery occlusion. These patients are at risk of further clot propagation or stump embolization to distal basilar artery branches; in addition, there is increased morbidity and mortality risk related to simultaneous cardiogenic emboli origin [31]. Small-vessel lacunar strokes of the lateral medulla may occur, but they are infrequent [1]. A thorough vascular evaluation to identify the specific mechanism of a stroke is necessary; upon diagnosis, confirmation is derived from imaging evidence of DWI-restricted diffusion. The combination of STA (ataxia type 3) central HINTS and OLD, as exemplified in cases 5, 6, 7, and 9 (Video S1), preceded the imaging evidence of LMS, as all four had initially false-negative MRIs. Central HINTS, STA, and OLD in these patients pointed to a lateral medulla stroke, confirmed by a subsequent MRI obtained 48 h later.

### 4.4. Strengths and Limitations of the Study 

The main value of this study is the simplicity of testing the OLD/STA combination in AVS patients with central HINTS. The busy frontline providers and neurology consultants require expeditious tests with high localizing value; this, of course, does not replace the complete neurological exam [21,32,33,34,35,36], but identifies patients that require an in-depth evaluation and perhaps those who need early intervention and close monitoring. The main limitation of the study is the fact that we did not include LMS patients who did not have AVS. In addition, detailed vascular imaging was not uniformly performed, and none of the patients in this cohort underwent thrombolysis. Even though all patients were evaluated within 24 h after symptom onset, their initial NIH stroke scale was 2 or 3, mostly because of STA. The normal head impulse, nystagmus, skew deviation, and OLD tests could add one additional point; therefore, in the absence of progression to suggest basilar artery occlusion, these patients did not receive treatment. 

Besides the highly lateralizing clinical signs described above, the presence of radiographic horizontal conjugate gaze deviation and loss of the normal signal void from the ipsilateral vertebral artery (vertebral artery target sign) [13] provide early additional evidence of LMS even when an overt LMS is not present. This information is potentially critical for rapid intervention when required (thrombolysis). The combination of clinical and early radiographic signs may offer a sensitive pro-thrombolytic intervention tool in the future. Larger LMS series may provide compelling support to implement thrombolysis based on clinical and early radiographic signs, even in the absence of a positive DWI scan, which remains the “gold standard signature of an acute stroke”.

**Figure 4:**
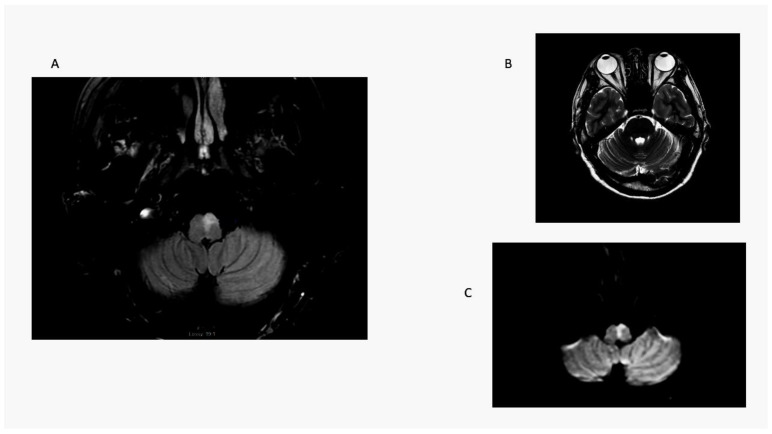
Medial medullary stroke. Panel (**A**): axial T2 MRI shows an elongated paramedian area of hyperintensity involving the left medullary pyramid and extending toward the dorsal medulla near the floor of the fourth ventricle. Note absence of radiographic ocular deviation in a T2 axial MRI (Panel (**B**)). Panel (**C**) is a DWI scan showing restricted diffusion confirmed by ADC map. Here, a combination of right-sided weakness and grade 3 ataxia causes contralesional falls.

Unlike LMS patients, the one medial medullary stroke (MMS) patient in this series had contralesional truncal ataxia and hemiparesis; he did not have OLD. He was not able to sit at the bedside and could not stand with a wide base initially (ataxia grade 3) (Figure 4). In a previous series, radiographic ocular deviation occurred in MMS and correlated with an ipsilesional horizontal nystagmus slow phase rather than OLD in one patient [37]. An earlier large series of MMS patients did not include OLD testing [38].

### 4.5. Future Studies

The pathogenesis of LMS and medulla strokes, in general, frequently involves large-vessel disease. In addition, the high frequency of unilateral vertebral artery hypoplasia [39] combined with the vertebral artery (V1 and V2) position within the lateral cervical vertebrae renders this vessel susceptible to arterial dissection. At the present time, besides basilar artery occlusion posterior, circulation stroke does not routinely undergo thrombolysis [40]. Only a handful of patients with Wallenberg’s syndrome have undergone thrombolysis [41]. Perfusion studies of the posterior fossa to identify the ischemic penumbra and infarct core (mean transient time or time to peak: T max and cerebral blood volume) have just begun [15]. The T max and cerebral blood volume values used in the anterior circulation will require validation for the posterior fossa. Technical limitations are challenging; however, in time, it is probable that technological advances now routinely available for anterior circulation will be available in identifying posterior fossa strokes that could benefit from thrombolysis. 

LMS Prognosis: the general consensus in the literature states that the short-term LMS prognosis for neurologic recovery enabling independent gait is favorable [1,42]. The prognosis in this series was also favorable and might depend, to some extent, on the presence of associated cerebellar infarcts.

In conclusion, regarding central HINTS, tests for STA and OLD examining AVS patients’ ability to sit without support are easy to implement and have significant localizing value to the lateral medulla, identifying patients that require an in-depth neurological examination. Even though LMS is not a frequent AVS cause, the combination of these two findings had 100% specificity in this series, underscoring their diagnostic value. Management of the vascular cause is variable depending on etiology, atherosclerosis, extra or intracranial dissection, hypoplastic vertebral artery, and vasculitis. Development of an ischemic posterior fossa index that takes into consideration the physical findings noted in this paper and the introduction of effective vascular imaging and perfusion studies may bring the management of brainstem and cerebellar strokes up to the current standard for ischemic anterior circulation stroke.

## Figures and Tables

**Table 1:** Ocular lateropulsion (OL), radiographic gaze deviation, and degree of truncal ataxia.

Case	Radiographic H CGD and Type of Corrective Centripetal Saccades	Lesion Location	Nystagmus	H HIT	Skew	Timing and Duration of OL	Truncal Ataxia Grade
1 R OLD 52	RE: 32.2 LE: 32.2 Hypometric saccades	R lateral medulla, cerebellar tonsil, uvula, and nodulus.	H direction changing	Normal	No	Noted at 32 h exam; present 6 weeks later. Resolved between 6 weeks and 5 months	Ataxia grade 3 to right
2 R OLD 75	RE: 21.9; LE 22.4. Hypometric saccades	R lateral medulla and lateral cerebellum.	H second degree to L	Normal	Ocular tilt reaction	Noted at 30 h exam; present during hospital stay for 3 days.	Ataxia grade 3 to right
3 L OLD 88	RE: 22.4; LE 26.6 Single rapid corrective saccades	L lateral pons, mid basilar stenosis.	H second-degree L	Abnormal	No	Noted at 24 h exam. Present for 5 days in ICU until cardiac arrest.	Ataxia grade 2 to left
4. L OLD 61	RE: 25.9; LE:30.3 Hypometric saccades	L lateral medulla.	H second-degree R	Normal	Yes	Noted at 72 h exam.	Ataxia grade 3 to left
5. R OLD 41	RE: 34.9; LE 25 Hypometric saccades	False-negative in initial MRI. Second MRI: stroke R lateral medulla.	Primary gaze h LBN. Did not follow Alexander’s Law	Normal	Yes	Noted at first exam and at 12 h. Resolved in 48 h.	Ataxia grade 3 to right
6. L OLD 49 F	RE: 21.4; LE: 36.2 Hypometric saccades	False-negative in initial MRI. Second MRI: L lateral medulla.	H 1st-degree RBN	Normal	Yes	Not checked at first visit; present at 36 h exam; lost to f/u.	Ataxia grade 2 to left
7. R OLD 62 F	RE: 13.6; L:0.1 Hypometric Saccade	False-negative in initial MRI. Second MRI: stroke R lateral medulla.	H LBN second degree	Normal	yes	OL Noted at 24 h exam, resolved in 12 h.	Ataxia grade 3 to right
8 R OLD 63 M	RE: 29.3; LE: 27.35	R lateral medulla.	Second-degree torsional to L shoulder/h-RBN	Normal	OTR	Noted at 32 h exam.	Ataxia grade 3 to right
9 R OLD 51 M	RE: 35.6; LE 26.6	False-negative in initial MRI. Second MRI: stroke R lateral medulla.	UBN	Normal	yes	Noted at 48 h exam.	Ataxia grade 3 to right
10 R OLD 59 M	RE: 45.3, LE: 42.3	R dorsolateral medulla.	Bilateral h gaze-evoked nystagmus	Normal	Yes	OLD at 6 h	Ataxia grade 2 to right
11 R OLD 28 M	RE: 35.6, LE: 20.9	R lateral medulla and cerebellum.	H LBN torsional top pole to left shoulder.	Normal	No	OLD at 24 h.	Ataxia grade 3 to right
12 L OLD 39 M	RE: 22.4, L: 18/1	L lateral medulla and cerebellum.	H RBN in center fixation, h-gaze-evoked nystagmus.	Normal	No	OLD at 24 h.	Ataxia grade 3 to left
13. R OLD 45 M	RE:40, LE: 34.4	R lateral medulla.	No nystagmus.	Normal	No	OLD at 6 h.	Ataxia grade 3 to right

## Data Availability

Data availability can be shared upon request.

## Supplementary Materials

The following supporting information can be downloaded at: https://www.mdpi.com/article/10.3390/audiolres13050068/s1, Video S1: Compressed grade 3 truncal.mp4; Video S2: Compressed Severe Truncal Ataxia.mp4.
